# Thoracic perspective revisited in chronic liver disease

**DOI:** 10.1093/gastro/gov017

**Published:** 2015-05-11

**Authors:** Binit Sureka, Kalpana Bansal, Yashwant Patidar, Sachin Kumar, Ankur Arora

**Affiliations:** ^1^Department of Radiology/Interventional Radiology and; ^2^Department of Pulmonary Medicine, Institute of Liver and Biliary Sciences, New Delhi, India

**Keywords:** cirrhosis, infection, portosystemic, portopulmonary, computed tomography

## Abstract

A variety of chest manifestations are seen in patients with chronic liver diseases, namely hepatopulmonary syndrome, portopulmonary hypertension, intrathoracic portosystemic collaterals, hepatic hydrothorax, infections, drug-induced changes, manifestations of hepatocellular carcinoma, gynecomastia, acute respiratory distress syndrome, autoimmune changes, aspiration pneumonitis and changes due to α_1_-antitrypsin deficiency. Gastroenterologists and radiologists should be aware of these entities; knowledge of the imaging findings specific to each condition is of prime importance for managing such patients.

## Introduction

Liver cirrhosis is a slowly progressing disease characterized by replacement of healthy liver parenchyma by scar and fibrotic tissue. Chronic infection with hepatitis B virus, hepatitis C virus (HCV) and alcohol consumption are the leading causes of cirrhosis worldwide. Viral hepatitis is the leading cause of cirrhosis in developing countries, and alcohol, HCV and non-alcoholic steatohepatitis are the most significant causes of cirrhosis in the developed countries. The economic burden due to cirrhosis is high, with current statistics of approximately 31 000 deaths each year in United States. HCV infection is more prevalent, with an average of 3.3 million people being chronically infected. Approximately 16 000 people die of hepatitis C in the United States, and around 3000 deaths have been reported annually due to hepatitis B infection. Liver disease is the fifth major killer in the United Kingdom, and each year around 7000 people die from cirrhosis. Hepatitis B is endemic in China, and 300 000 people die from hepatitis B-related diseases every year. According to experts, India will become the “world capital of liver disease” by 2025 [1, 2]. The direct health care costs associated with cirrhosis are high. According to the US Centers for Disease Control and Prevention, the national costs for treating cirrhosis in 2008 ranged from $14 million to $2 billion [3].

Different chest complications may occur in patients with chronic liver disease (Table 1

**Table 1.** Chest manifestations in cirrhosis

Pulmonary vascular complications
Hepatopulmonary syndrome (HPS) Portopulmonary hypertension (PPH)
Pleural effusion
Hepatic hydrothorax Subacute bacterial pleuritis/empyema (SBP)
Intrathoracic portosystemic collaterals
Lung injury in liver failure
Acute respiratory distress syndrome (ARDS) Aspiration pneumonia Volume overload Pulmonary edema
Infections
Bacterial, fungal, viral Tubercular
Intrathoracic manifestations of hepatocellular carcinoma
Pulmonary metastases Lymph nodal metastases Tumor thrombus in IVC/right atrium Pulmonary tumor emboli
Drug induced
Interferon-induced sarcoidosis (in hepatitis C) Methotrexate pneumonitis
Gynecomastia
Cause-specific pulmonary complications
Alpha-1 antitrypsin deficiency Cystic fibrosis
Inflammatory and autoimmune associations
Interstitial lung disease (primary biliary cirrhosis)
α_1_-antitrypsin deficiency related
Panacinar emphysema Bronchiectasis
Muscle wasting/Sarcopenia
Tense ascites

). In this article, we will focus on various thoracic complications associated with cirrhosis.

## Hepatopulmonary syndrome

Hepatopulmonary syndrome (HPS) is seen in 4–29% of patients with liver cirrhosis [4]. It is a triad comprising liver disease, increased alveolar arterial oxygen gradient while breathing room air (arterial hypoxemia) and intrapulmonary vascular dilatations. This syndrome manifests clinically as progressive dyspnea, cyanosis and clubbing in a known patient with cirrhosis. Long-term survival for all HPS patients in general is worse for those with lower baseline PaO_2_ (50 mmHg). HPS is an indication for orthotopic liver transplantation (OLT) whatever the severity of hypoxemia. However, besides the favorable long-term survival of HPS patients with OLT have a high postoperative mortality (mostly within 6 months) [5–7]. At an observation period of 2.5 years, the mortality rate for HPS patients is approximately 40–63%. The leading cause of death is hemorrhagic shock secondary to gastrointestinal bleeding [8].

The risk seems to be highest in Child C liver patients. Pathophysiology underlying this vasodilatation is excessive production of vasodilators, particularly nitric oxide, tumor necrosis factor alpha and heme oxygenase-derived carbon monoxide [9]. In the presence of portal hypertension, hepatic production of endothelin-1 and expression of endothelial type B receptors occur, but no type A receptors increase in pulmonary vasculature. This leads to increased signaling and production of nitric oxide with the overall effect of pulmonary vascular dilatation, which is pathognomonic of hepatopulmonary syndrome.

**Figure 1.**
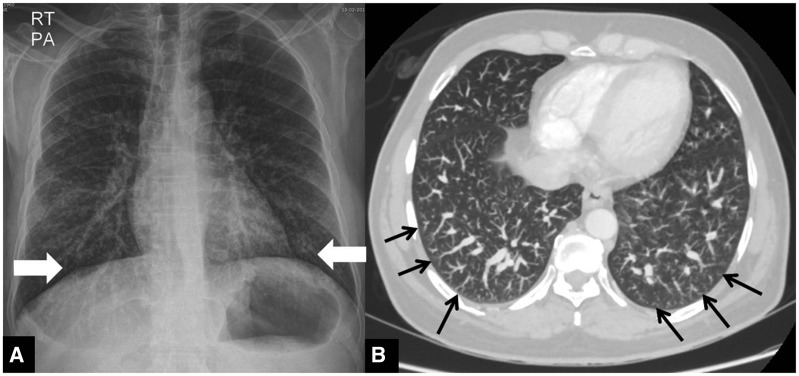
(A) Hepatopulmonary syndrome. Chest radiograph of a case of type 1 hepatopulmonary syndrome in a man with liver cirrhosis showing increased bronchovascular markings in bilateral lower zones (arrows). (B) CT coronal maximum intensity projection image of lung window showing dilated distal pulmonary arteries with subpleural telangiectasia (arrowheads).

On imaging, two types of patterns can be seen. Type 1 is the most common and is seen in nearly 86% of the cases. The hallmark on plain chest radiographs is nodular or reticulonodular opacities in lower zones. On computed tomography (CT), dilated pulmonary vessels are seen in subpleural location with subpleural telangiectasia. These vessels do not taper and extend to the peripheral pleural surface of the lungs (Figure 1). Type 2 is less common and is characterized by the presence of large arteriovenous malformations or nodular dilatation of peripheral pulmonary vessels, which are connected by a feeding artery and draining vein as viewed on CT scan [10].

## Portopulmonary hypertension

Portopulmonary hypertension is defined as pulmonary artery hypertension that develops in a situation of portal hypertension. Liver and extra-hepatic causes that can cause portal hypertension are a predisposing factor for the development of portopulmonary hypertension. Thus, portal hypertension seems to be the required driving force of pulmonary hypertension. Portopulmonary hypertension is seen in 2–5% of patients with liver cirrhosis [11]. Criteria for labeling the patient as having features of portopulmonary hypertension are mean pulmonary artery pressure >25 mmHg at rest, increased pulmonary vascular resistance and pulmonary capillary wedge pressure <15 mmHg with features of portal hypertension [11]. There are three main etiopathogeneses for the development of portopulmonary hypertension: (i) release of vasoactive substances (serotonin, interleukin 1, endothelin 1 thromboxane), which can lead to vasoconstriction in pulmonary arteries; (ii) venous thromboembolism as blood clots from portal vein can pass through portosystemic shunts and reach the pulmonary circulation, causing pulmonary hypertension; and (iii) high cardiac output associated with cirrhosis exposing the pulmonary vascular bed to increased shear stress and leading to hypertrophy and proliferation of pulmonary arterial endothelial cells and ultimately to vasoconstriction. The development of severe pulmonary hypertension in patients who have cirrhosis is an ominous prognostic sign. Clinically, the patient typically presents with progressive dyspnea on exertion. Other less common symptoms are fatigue, palpitations, syncope or chest pain.

From the pathophysiological aspect, hepatopulmonary syndrome and portopulmonary hypertension are complete opposites. Hepatopulmonary syndrome is due to vasodilatation, whereas portopulmonary syndrome is due to vasoconstriction.

**Figure 2.**
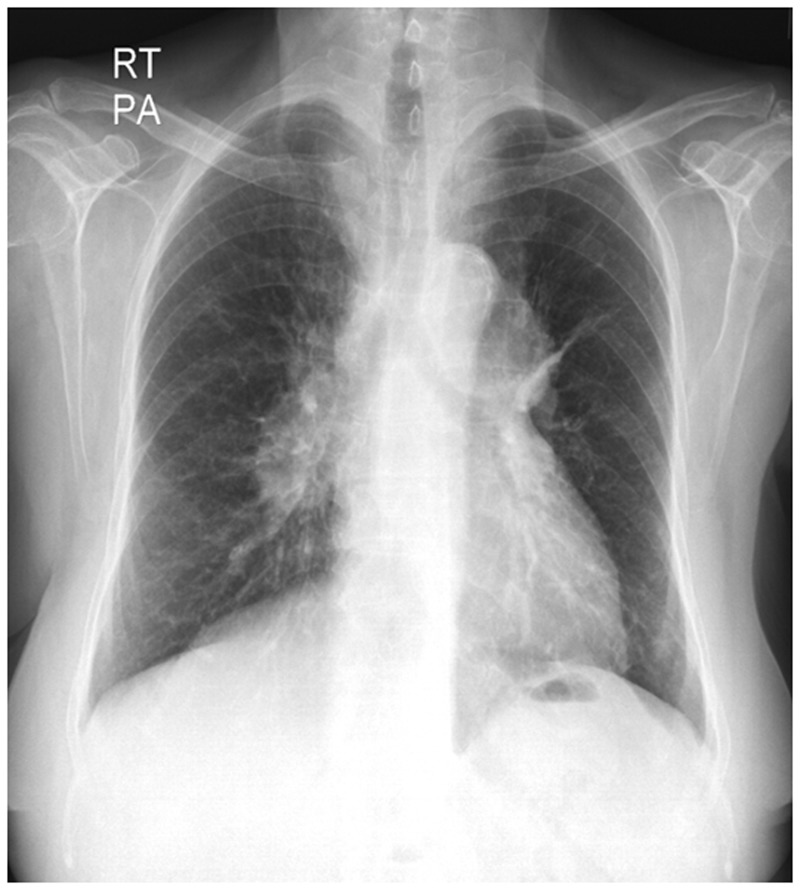
Portopulmonary hypertension. Chest radiograph of a case of portopulmonary hypertension in a patient with cirrhosis showing dilated central pulmonary arteries and pruning of peripheral pulmonary vessels suggestive of pulmonary arterial hypertension.

On imaging, chest radiographs show classical features of pulmonary arterial hypertension, namely prominent central pulmonary arteries, pruning of peripheral pulmonary vessels, elevated cardiac apex due to right ventricular enlargement and right atrial enlargement (Figure 2). CT shows dilated main pulmonary artery (>29 mm or segmental artery-to-bronchus ratio >1:1 in three of four pulmonary lobes), ratio of the main pulmonary artery diameter to the aortic diameter >1, mosaic pattern of lung attenuation, right ventricular hypertrophy (wall thickness >4 mm), leftward bowing of interventricular septum and neovascularity (that do not conform to pulmonary arterial anatomy).

## Hepatic hydrothorax

**Figure 3.**
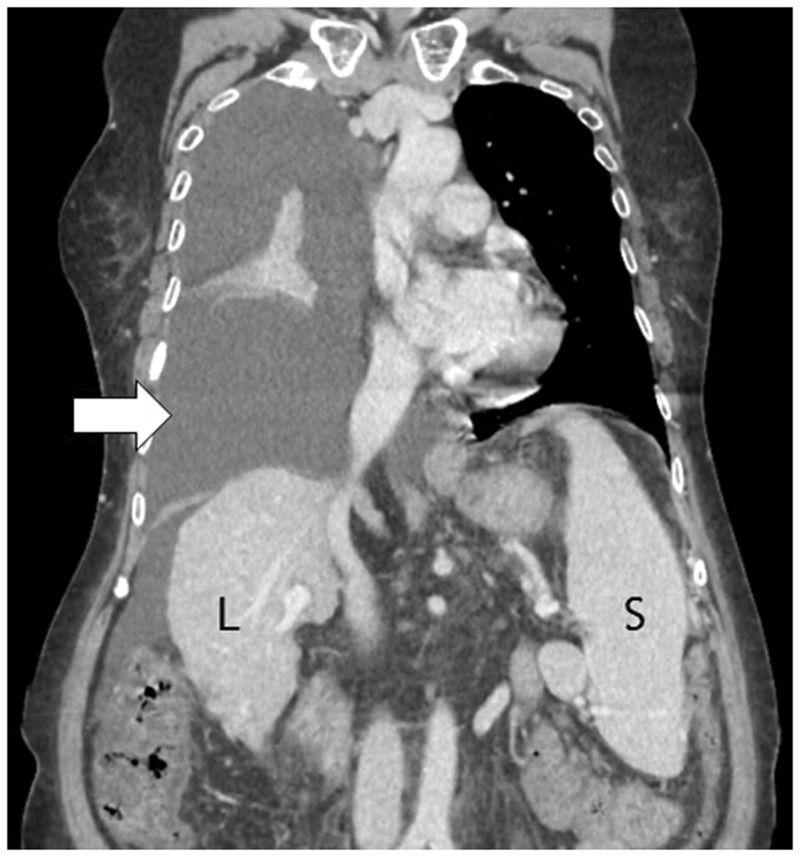
Hepatic hydrothorax. Coronal reformatted contrast-enhanced CT scan in a woman with liver cirrhosis showing gross right-sided pleural effusion (arrow). Also noted is presence of ascites, cirrhotic changes in liver (L) and splenomegaly (S).

Hepatic hydrothorax is seen in 5–10% of cirrhotic patients. It is defined as development of significant pleural effusion, which is usually right sided (>500 ml), in the absence of a cardiopulmonary cause in a cirrhotic patient with portal hypertension. The proposed etiopathogeneses are varied: decreased colloid osmotic pressure due to hypoalbuminemia, leakage of ascitic fluid via diaphragmatic defects (congenital defects or rupture of pleuroperitoneal blebs), transdiaphragmatic migration of fluid via lymphatic channels or azygous venous hypertension [12]. The effusion is usually right sided (Figure 3); however, it is seen on the left side in 13% of cases and bilaterally in 2% cases [10]. Biochemical analysis of pleural fluid reveals a transudative nature.

## Subacute bacterial empyema

**Figure 4.**
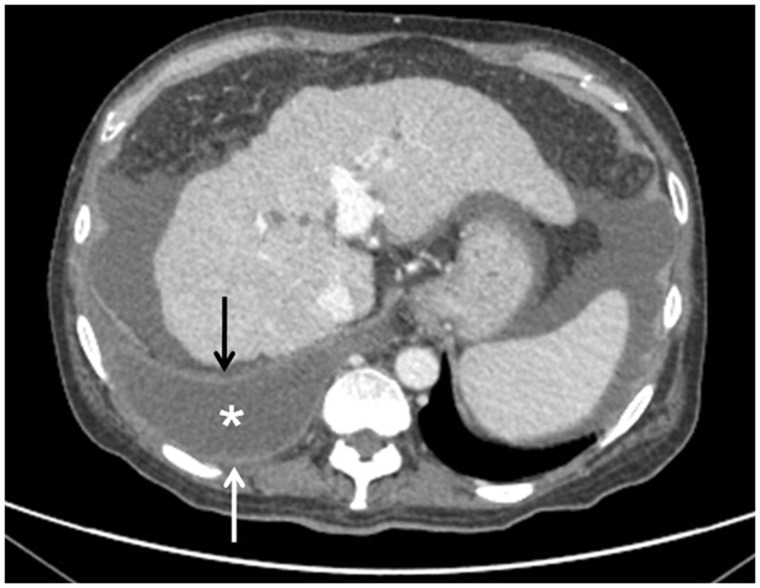
Subacute bacterial empyema. Axial contrast-enhanced CT scan in a known case of cirrhosis showing empyema (asterisk) in right pleural cavity with enhancement of pleura (arrows). Ascites and changes of cirrhosis in liver are also noted.

Spontaneous bacterial empyema can develop in patients with hepatic hydrothorax and requires a high index of clinical suspicion (Figure 4). Clinically, the patient has fever, pleuritic chest pain, deterioration of clinical status or encephalopathy. It is diagnosed when either the polymorphonuclear cell count > 500 cells/mm^3,^ or the pleural fluid shows positive culture with cell count > 250 cells/mm^3^ after excluding parapneumonic effusion. The microorganisms involved are *Escherichia coli, Streptococcus, Enterococcus, Klebsiella* and *Pseudomonas* [12].

## Intrathoracic portosystemic collaterals

Varices are known to develop in patients having cirrhosis with portal hypertension. These varices develop due to enlargement of pre-existing anastomosis between the portal and systemic venous systems. There could be esophageal, paraesophageal or cardiophrenic varices depending on their location [10].

**Figure 5.**
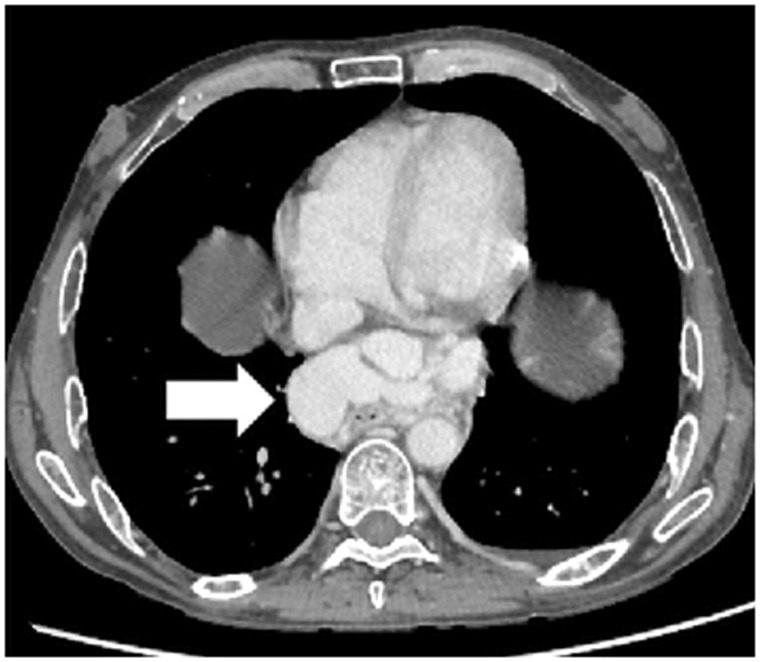
Intrathoracic portosystemic collaterals. Axial contrast-enhanced CT scan showing intrathoracic portosystemic collateral in the form of dilated paraesophageal veins (arrow) in a case of cirrhosis.

On imaging, esophageal varices are seen as nodular thickening of the esophageal wall or enhancing nodular lesions protruding into the esophageal lumen. Paraesophageal varices are seen as enhancing nodular vascular channels causing lateral bulging of paraspinal interfaces, or they may cause obliteration of the azygoesophageal recess and descending thoracic aortic interface (Figure 5). Cardiophrenic angle varices are the least common and consist of dilated pericardiacophrenic veins. A tortuous vascular channel is seen communicating the left hepatic vein with the inferior phrenic vein and pericardiophrenic vein and ultimately draining into the left innominate vein. These are usually seen in patients with cirrhosis caused by membranous obstruction of the inferior vena cava.

## Acute respiratory distress syndrome

According to the American-European Consensus Conference (AECC) definition [13] published in 1994, acute respiratory distress syndrome (ARDS) is defined as the acute onset of respiratory failure, bilateral infiltrates on chest radiograph, hypoxemia as defined by a PaO_2_/FiO_2_ ratio ≤ 200 mmHg and no evidence of left atrial hypertension or pulmonary capillary pressure <18 mmHg (if measured) to rule out cardiogenic edema. Acute lung injury, the less severe form of acute respiratory failure, is different from ARDS by the degree of hypoxemia; in fact, it is defined by a 200 mmHg < PaO_2_/FiO_2_ ≤ 300 mmHg. A draft definition proposed three mutually exclusive categories of ARDS based on degree of hypoxemia: mild (200 mmHg < PaO_2_/FIO_2_ ≤ 300 mmHg), moderate (100 mmHg < PaO_2_/FIO_2_ ≤ 200 mmHg) and severe (PaO_2_/FIO_2_ ≤ 100 mmHg) and four ancillary variables for severe ARDS: radiographic severity, respiratory system compliance (≤40 mL/cmH_2_O), positive end-expiratory pressure (≥10 cmH_2_O) and corrected expired volume per minute (≥10 L/min) [14].

Chronic liver disease acts as a comorbid condition in the development of ARDS. It results from systemic spillover of pro-inflammatory substances due to changes in hepatic blood flow and its ability to clear the toxins or an imbalance in the level of Na^+^, K^+^-adenosine triphosphatase inhibitor, which is elevated in the serum of patients with fulminant hepatic failure [15]. ARDS carries high morbidity and mortality once it has set in.

**Figure. 6.**
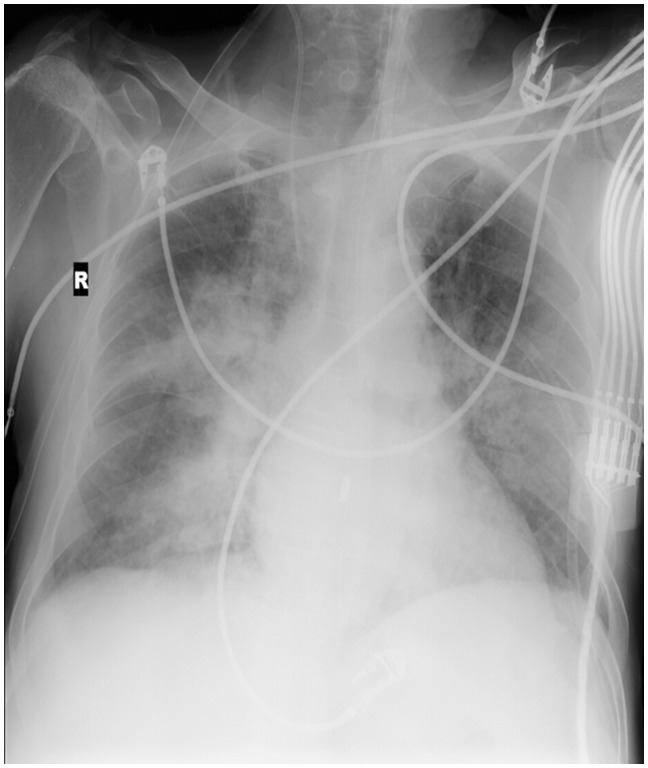
Acute respiratory distress syndrome. Chest radiograph in a patient with acute respiratory distress syndrome (admitted to intensive care unit) showing diffuse coalescent opacities and consolidation in both lungs.

**Figure 7.**
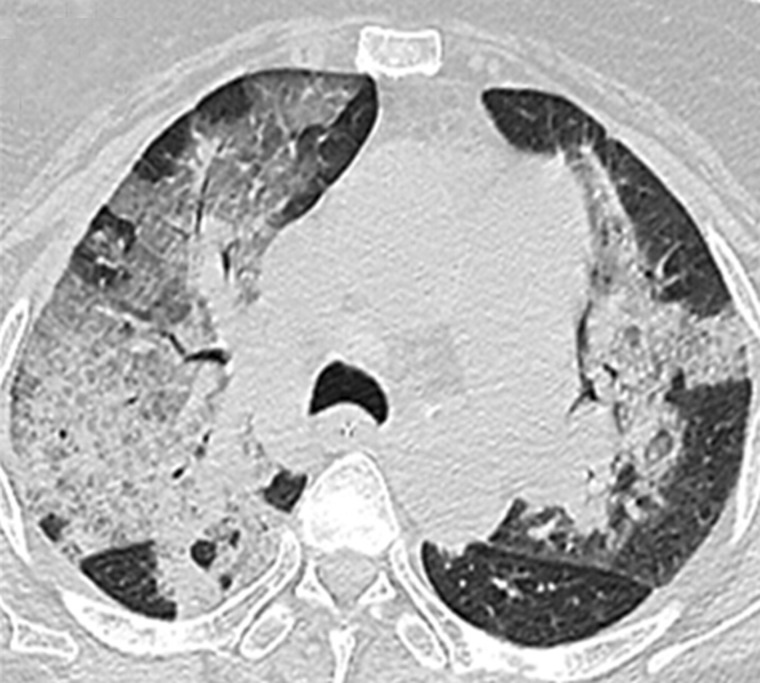
Pulmonary hemorrhage. Axial high resolution CT scans showing consolidation superimposed with ground glass opacities suggestive of pulmonary hemorrhage.

ARDS is difficult to differentiate from pulmonary edema and pulmonary hemorrhage on imaging. Patchy coalescent opacities and diffuse bilateral consolidation are seen on chest radiographs (Figure 6). CT shows features of dependent dense consolidation and diffuse bilateral ground-glass opacities (Figure 7).

## Infection

**Figure 8.**
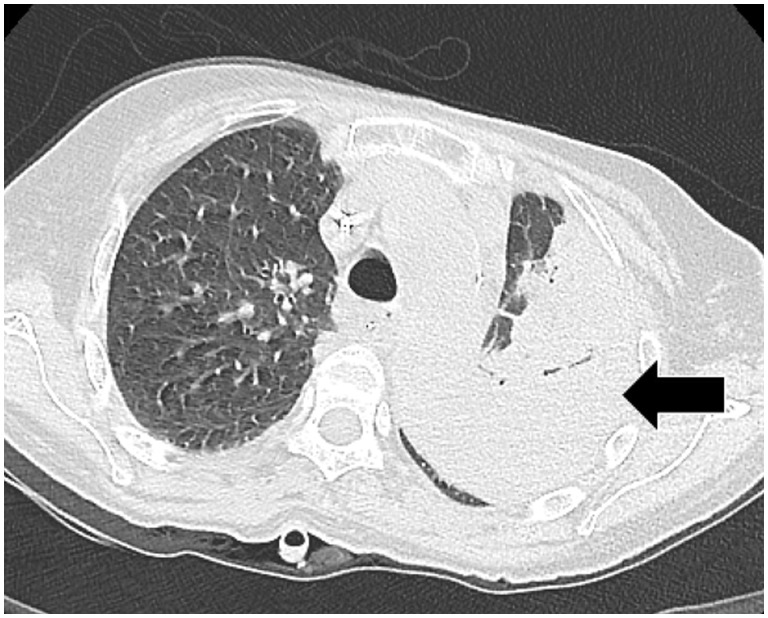
Pneumonia. Axial CT scan lung window section showing left lung consolidation (arrow). Sputum culture demonstrated S*taphylococcus aureus.*

**Figure 9.**
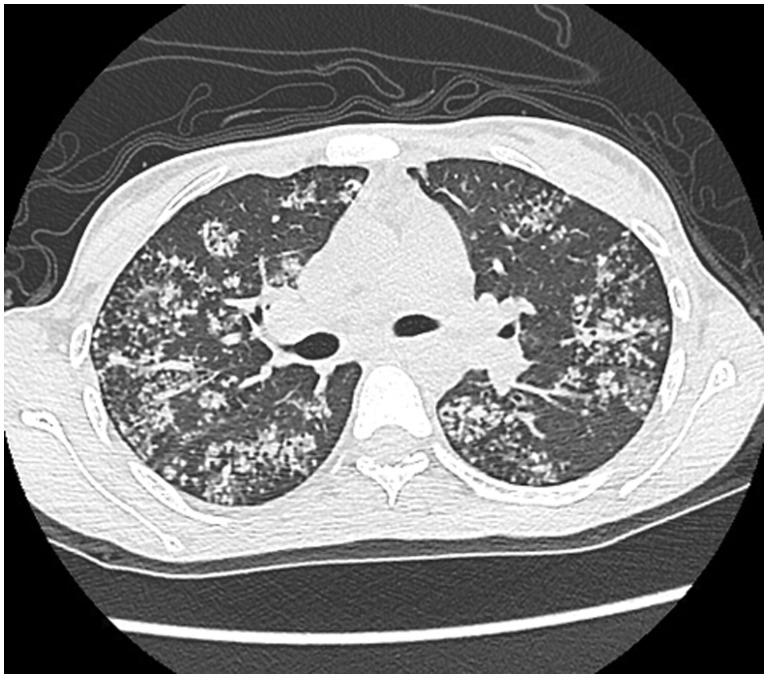
Pulmonary tuberculosis. High-resolution CT scan of lung window showing pulmonary tuberculosis in a case of cirrhosis. Centrilobular nodules are seen in both lungs with tree-in-bud appearance.

Patients with chronic liver disease have a compromised immune status that predisposes them to increased risk of pulmonary infections by a variety of bacterial, fungal and viral infectious organisms. Decompensated cirrhosis has more frequent episodes of infections than compensated cirrhosis. The most common infections in cirrhotics are spontaneous bacterial peritonitis (25%), followed by urinary tract infection (20%), pneumonia (15%), bacteremia following a therapeutic procedure, cellulitis and spontaneous bacteremia. Community-acquired infections are the most frequent, although hospitalized patients admitted to intensive care units have a high incidence of nosocomial pneumonias due to predisposing factors such as tracheal intubation, esophageal tamponade or hepatic encephalopathy. The most common bacterial microorganism involved is *Streptococcus pneumoniae.* Others agents are *Haemophilus influenzae*, *Pseudomonas aeruginosa, Klebsiella pneumoniae* and Mycoplasma and Legionella species*.* Hospital-acquired pneumonia is predominantly caused by Gram-negative bacilli and staphylococci (Figures 8 and 9) [16].

Tuberculous infection in patients with cirrhosis appears as extrapulmonary involvement more frequently than other infections. Ascites due to peritoneal tuberculosis may be difficult to diagnose in the setting of liver cirrhosis, where portal hypertensive ascites is common. Laboratory ascitic fluid analysis is recommended in cases that demonstrate raised adenosine deaminase levels, total lymphocyte count > 500 with predominant lymphocytes and protein >2.5 gm% in tubercular ascites.

**Figure 10.**
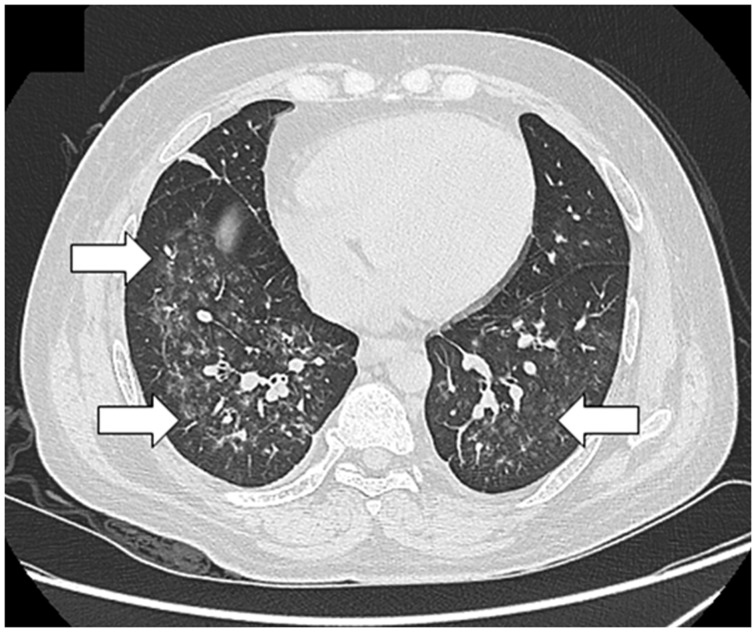
*Pneumocystis jirovecii* pneumonia. High-resolution CT scan of a lung window in a case of *Pneumocystis jirovecii* pneumonia showing bilateral perihilar ground glass opacities and few centrilobular nodules.

Fungal infections, especially *Candida* species, are involved in up to 15% of severe sepsis in cirrhosis [17]. Other organisms are *Aspergillus fumigatus* and *Pneumocystis jirovecii* (Figure 10). Infections with multiple fungal agents coexisting in the same patient have also been described [17].

Viral lung infections can also be caused by *Cytomegalovirus* (CMV) in patients with cirrhosis due to immunocompromised status. A high index of suspicion is required for establishing the diagnosis of CMV pneumonia. Diagnosis depends upon radiological evidence together with bronchoalveolar lavage culture or histopathological evidence of CMV-induced changes in lung. On imaging, ground glass attenuation, consolidation, discrete pulmonary nodules or masses are seen. Atypical patterns are nodules with halo, peribronchovascular thickening, bronchiectasis, pleural effusion and nodules with tree-in-bud [18].

## Intrathoracic manifectations of hepatocellular carcinoma

Any cirrhotic liver is predisposed to developing hepatocellular carcinoma (HCC). HCC is most prevalent in those infected with HCV infection (17–30%), followed by hereditary hemochromatosis (21%), hepatitis B virus infection (10–15%), alcoholic cirrhosis (8%) and advanced biliary cirrhosis (4%) [19].

**Figure 11.**
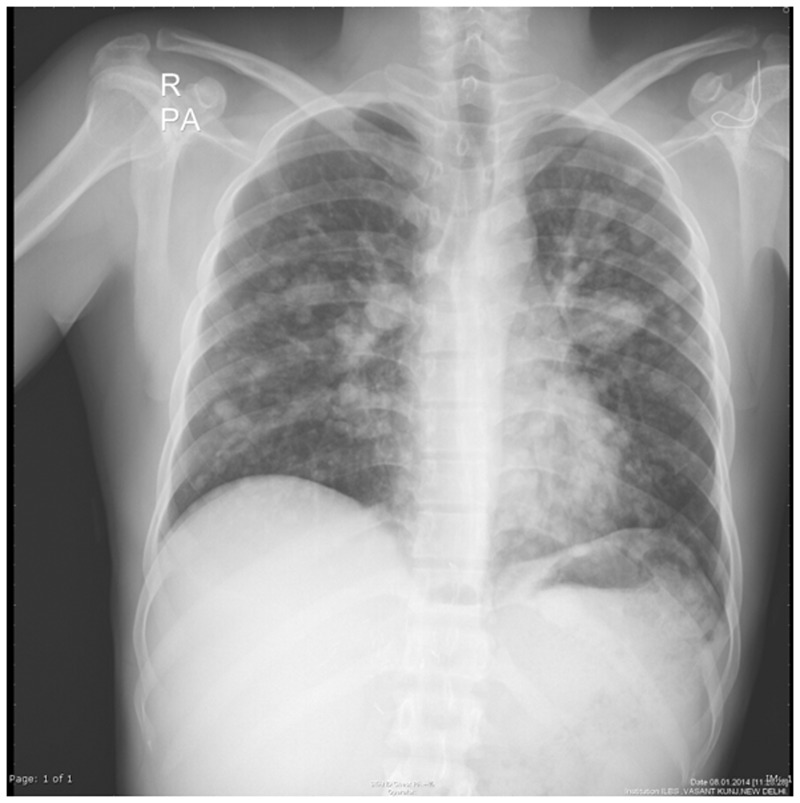
Metastases. Chest radiograph in a 50-year-old man with liver cirrhosis and hepatocellular carcinoma showing multiple nodular opacities in bilateral lungs, suggestive of hematogenous metastases.

Intrathoracic manifestations of HCC are pulmonary metastasis and metastatic lymphadenopathy (Figure 11). Other manifestations are pulmonary tumor emboli and tumor extension into the inferior vena cava or right atrium.

## Drug-induced pulmonary complications

Sarcoidosis is a rare complication of interferon therapy. The exact mechanism by which interferon induces sarcoidosis is unknown. Studies indicate that the activation of macrophages and uncommitted differentiation of CD4-positive T cells into Th1 effector cells leads to unregulated production of interferon, which is responsible for the disease [20, 21].

**Figure 12.**
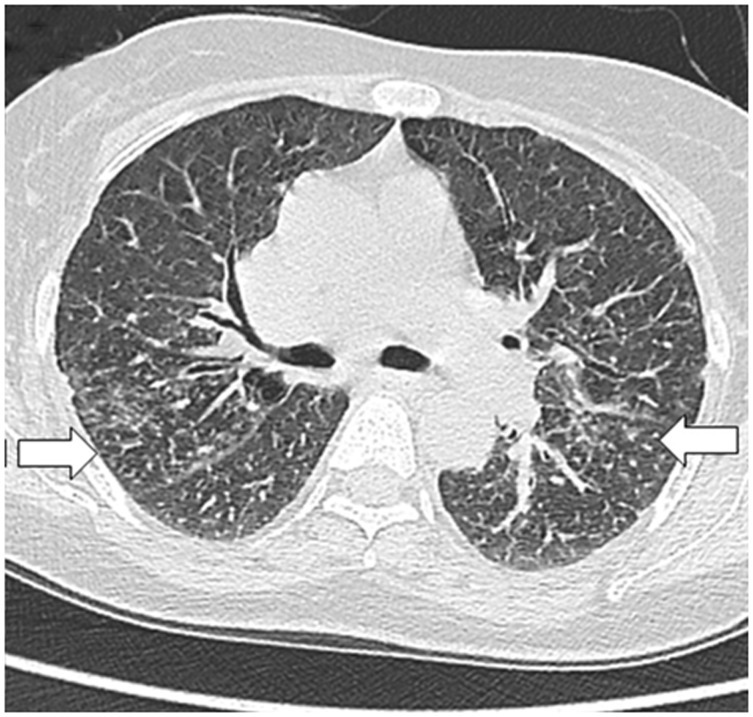
Sarcoidosis. High-resolution CT scan showing numerous tiny lung nodules with a perilymphatic and interlobular septal distribution (arrow).

Although any organ system can be involved, the lung and mediastinal lymph nodes are the most frequent sites of disease. On chest radiographs, sarcoidosis is seen as bilateral hilar and right paratracheal lymphadenopathy. Lung changes may be manifested in the form of fine miliary opacities, reticular, reticulonodular or, less commonly, air-space opacities that are usually confined to the mid and upper lungs. On high -resolution CT, nodules classically show perilymphatic distribution (Figure 12) [22].

## Gynecomastia

In cirrhosis, the liver's ability to synthesize testosterone and metabolize estrogen is impaired, leading to a higher estrogen-to-testosterone ratio. Gynecomastia is a condition characterized by benign enlargement of breast tissue in males. Three patterns have been recognized: nodular, dendritic and diffuse glandular [23].

## Aspiration pneumonitis

Aspiration pneumonitis is seen in patients with hepatic encephalopathy due to variceal hemorrhage or during endoscopic interventions in these patients. On chest radiograph, airspace opacities are seen in lobar or segmental distribution. On CT, posterior segments of upper lobes and superior segments of lower lobes are most commonly involved.

## Inflammatory and autoimmune associations

**Figure 13.**
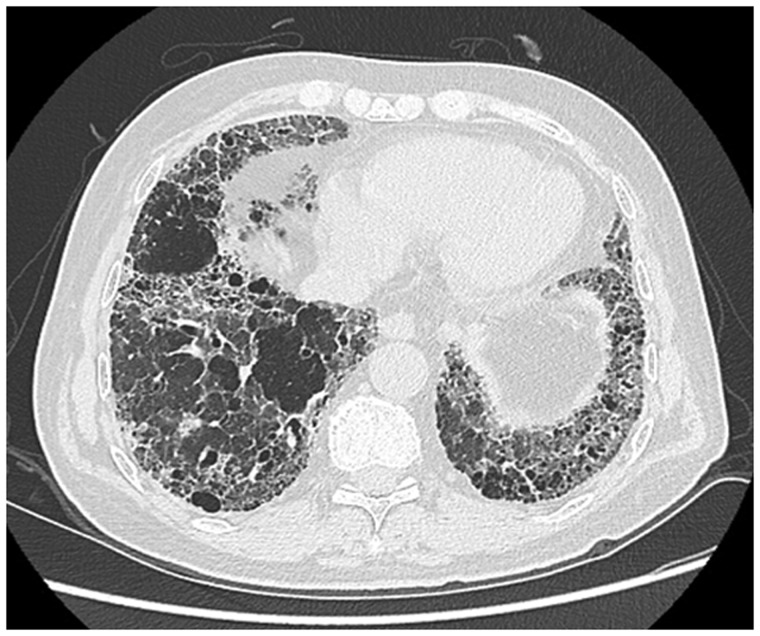
Idiopathic pulmonary fibrosis. High-resolution CT scan showing diffuse areas of interstitial thickening in both lungs with honey-combing and fibrosis in a case of biliary cirrhosis.

Primary biliary cirrhosis is a chronic autoimmune disorder of the liver characterized by slow progressive destruction of the bile ducts. The pulmonary manifestations that may be associated are pleural effusions, lymphocytic interstitial pneumonitis, sarcoidosis, pulmonary fibrosis (Figure 13), intrapulmonary granulomas, cryptogenic organizing pneumonia, obstructive airways disease, pulmonary hypertension, hepatopulmonary syndrome and pulmonary hemorrhage.

## Muscle wasting/sarcopenia

The most widely studied complications in cirrhotic patients until now were ascites, hepatic encephalopathy, variceal bleeding, kidney dysfunction and hepatocellular carcinoma; however, sarcopenia—or severe muscle wasting—one of the most common and frequently hidden complications that negatively impact survival and quality of life [24, 25]. Patients with advanced liver cirrhosis usually have severe protein wasting and loss of muscle mass. This muscle mass loss can affect both peripheral as well as the respiratory muscles and can contribute to chronic dyspnea in cirrhotic patients.

## Tense ascites

Dyspnea is a frequent complaint of patients with cirrhosis. Tense cirrhotic ascites causes respiratory difficulty and shortness of breath due to limited venous return from the lower limbs (pressure on the inferior vena cava) and impaired expansion of the lungs (elevation and mechanical pressure on the diaphragm) [26,27].

## Conclusion

Chronic liver disease can have a diverse range of thoracic manifestations. Other complications of liver disease that can cause dyspnea include anemia, hypoxemia and cirrhotic cardiomyopathy. Radiologists, gastroenterologists and other specialists should be aware of various intrathoracic manifestations that can be encountered while reporting these cases. Close correlation with the clinical picture, laboratory parameters and imaging findings will help in reaching the accurate diagnosis.

*Conflict of interest statement*: none declared.
